# Multimodality Imaging in Transcatheter Mitral Interventions

**DOI:** 10.3389/fcvm.2021.638399

**Published:** 2021-02-23

**Authors:** Evgenia Nikolou, Rajdeep Bilkhu, Tahir S. Kafil, Camelia Demetrescu, Prasanti Alekhya Kotta, Gianluca Lucchese, Nikolaos Tzemos, Julia Grapsa

**Affiliations:** ^1^Department of Cardiology, St George's University Hospitals NHS Foundation Trust, London, United Kingdom; ^2^Department of Cardiothoracic Surgery, Guys and St Thomas NHS Hospitals Trust, London, United Kingdom; ^3^Department of Cardiology, Western University, London, ON, Canada; ^4^Department of Cardiology, Guys and St Thomas NHS Hospitals Trust, London, United Kingdom

**Keywords:** mitral valve, transcatheter replacement, multimodality imaging, transcatheter repair, echocardigraphy

## Abstract

Multimodality imaging is of imperative value for the planning and guidance of transcatheter mitral valve interventions. This review employs the value of different imaging modalities and future implications for clinical practice.

## Introduction

Transcatheter mitral interventions (TMVI) have exceeded the expectations oftechnical progress over the last decade. The recent explosion of technological advances that allows the personalized management of every patient with mitral valve disease, highlights the pivotal role of multimodality imaging in the decision making process. Specific challenges include the large spectrum of underlying pathology, the multidimensional feature of mitral annulus, the interaction with the left ventricle and its outflow tract, the constantly raising number of different devices which are available on the market, and the prevention of thromboembolism. Despite multiple challenges, the tremendous value of TMVI is offering a solution to these patients who almost a decade ago would be considered inoperable (1, 2).

### The Existing Landscape of TMVI

There are 5 different categories of TMVI: balloon valvotomy, mitral valve leaflet repair (edge to edge and artificial chords), transcatheter mitral valve replacement, mitral annuloplasty, and paravalvular leak closure.

**Figure 1:**
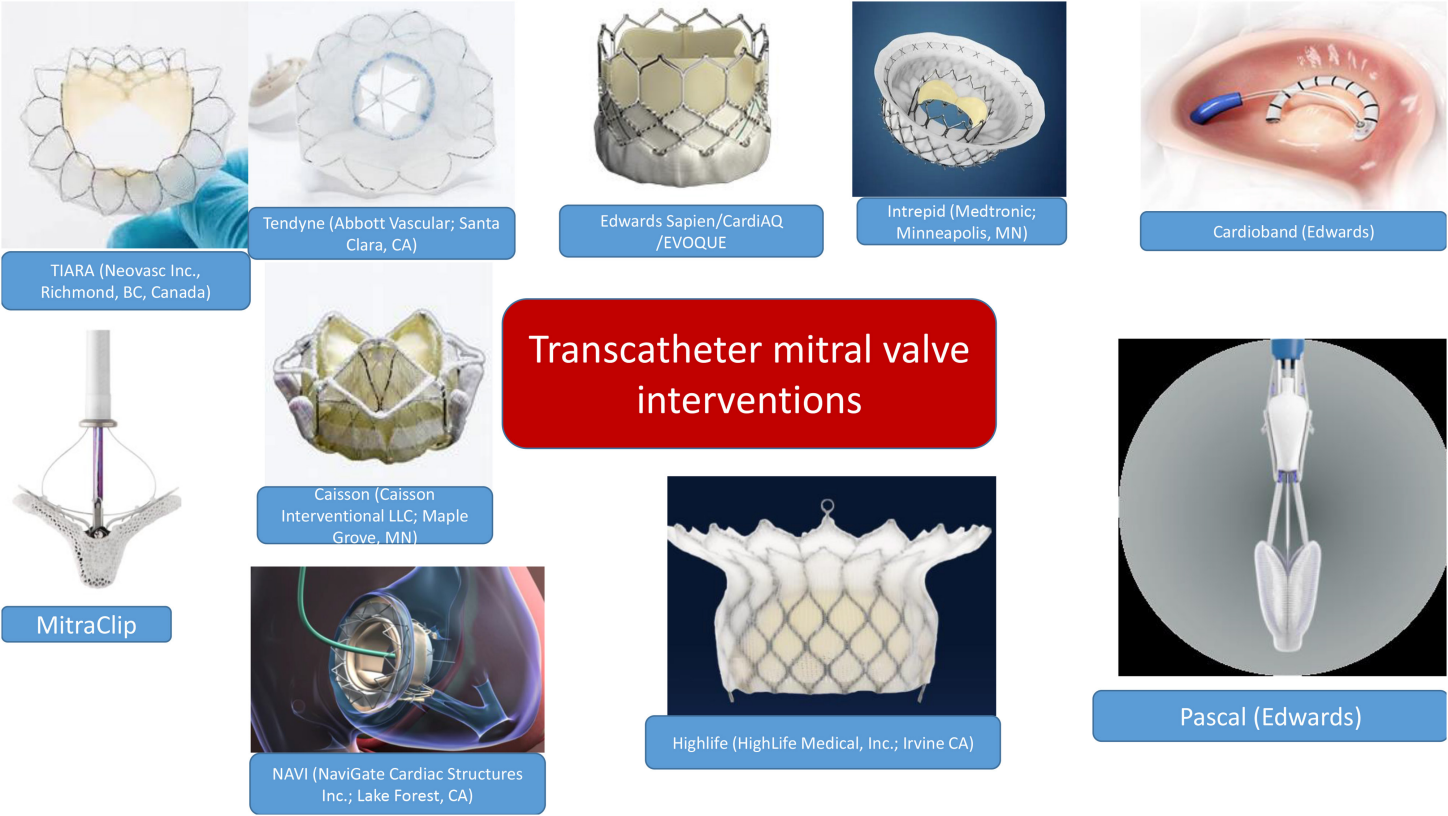
Transcatheter mitral valve interventions: available devices to date.

There has been a vast development of transcatheter valves which managed to address the issue of durability (3). Most important TMVI challenges were the adaptation to the oval and saddle-shape MV geometry, the non-rigid nature of the mitral annulus, the need to anchor the prosthesis, the large variation in mitral annular size, and the risk of LV outflow tract (LVOT) obstruction (4). Figure 1 summarizes the variety of devices currently available. Taking into consideration the scope of this review, we will focus on multimodality imaging rather than the technical description of transcatheter valves.

At the latest AHA/ACC guidelines on valvular heart disease (5), it is crystal clear that multidisciplinary decision between imagers, interventionists and surgeons is imperative to secure appropriate planning. In selected patients, hybrid mitral valve replacement may be a solution in a challenging scenario such as extensive mitral annular calcification (6).

## Multimodality Imaging

### Echocardiography

Transthoracic (TTE) and 3D transesophageal (TEE) echocardiography are the first line imaging tools for the assessment of mitral valve. For the purposes of this review, we will separate echocardiographic imaging into 3 parts: (i) pre-procedural, (ii) intra-procedural, and (iii) post-procedural.

(i) Pre-procedural

**Figure 2:**
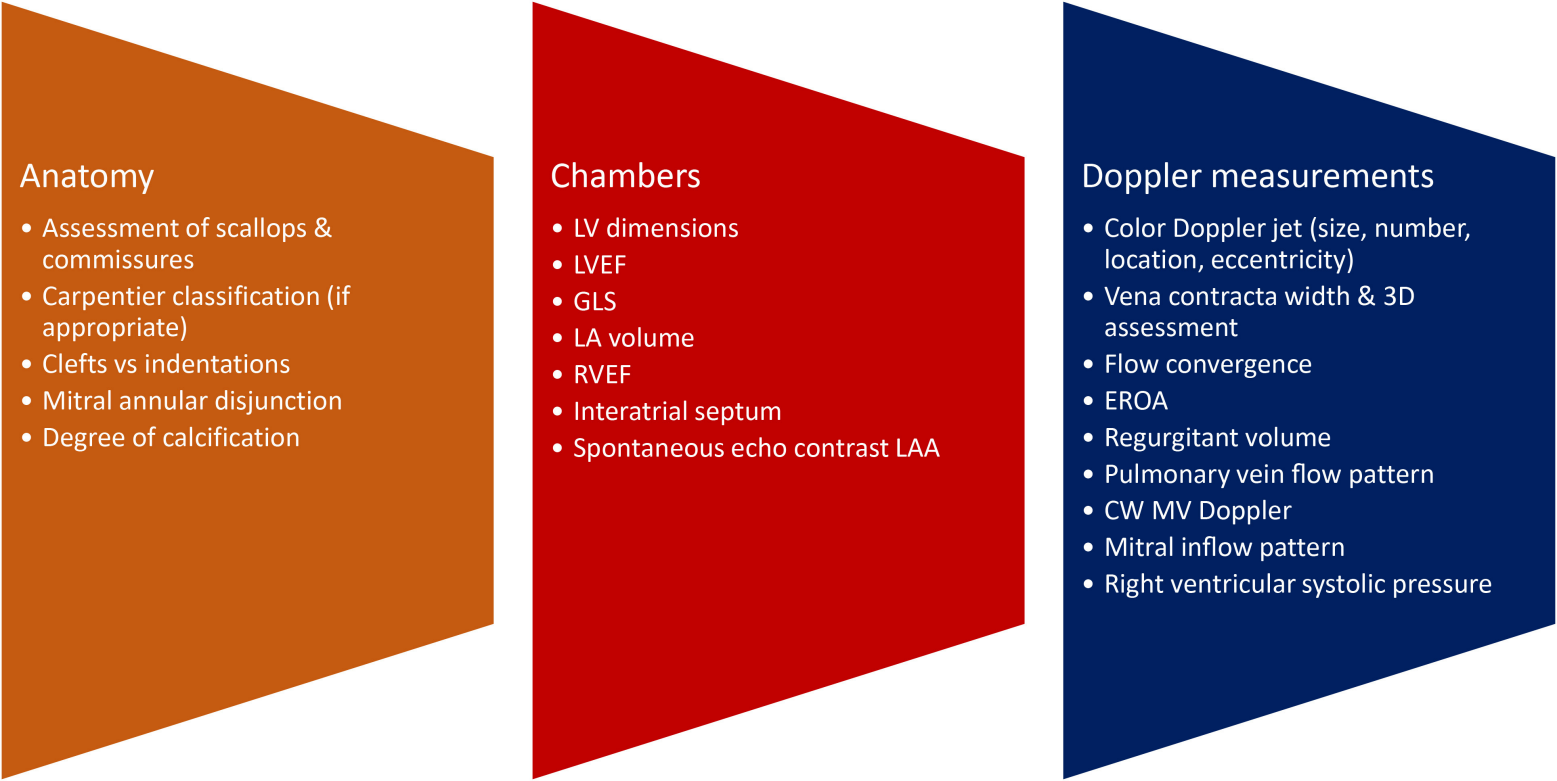
Pre-procedural assessment. LV, left ventricular; LVEF, left ventricular ejection fraction; GLS, global longitudinal strain; LA, left atrial; RVEF, right ventricular ejection fraction; LAA, left atrial appendage.

The role of the imager in guiding further management, in close collaboration with the interventionist and surgeon, is crucial. TTE as well as 3D TEE will identify the etiology and mechanisms of mitral pathology (Figure 2). It is important to define anatomy, the description of which is facilitated by the 3D TEE surgical view, highlighting the different anterior and posterior scallops as well as the commissures, the number of jets, the severity of valvular pathology, eccentricity, vena contracta, and flow convergence. In cases of doubtful mitral pathology, indirect signs such as pulmonary veins systolic flow reversal or the degree of tricuspid regurgitation (and subsequently the calculation of right ventricular systolic pressure) will further help in identifying the severity of the mitral valve disease. Furthermore, in the pre-procedural assessment, we evaluate left ventricular and atrial dimensions, ejection fraction (even if the latter is influenced by the volume overload) and the degree of elevated filling pressures. Ejection fraction as well as global longitudinal strain, as pre-procedural imaging indices, may be overestimated in cases of severe MR.

Technical considerations when obtaining the basic measurements for mitral valve assessment, such as the appropriate alignment of continuous wave Doppler with the jet and the appropriate settings for proximal isovelocity surface area (PISA) /EROA/regurgitant volume assessment, should be applied (7).

In addition, assessment of interatrial septum and the best anatomical position for transeptal puncture will facilitate the operator during TMVI. An incidental finding of a patent foramen ovale is of no hemodynamic significance. On the contrary, it may act in favor of the interventionist during the advancement of the guiding catheter.

For balloon valvotomy, the most common imaging tool for mitral stenosis assessment is the Wilkins score which consists of scoringleaflet mobility, valve and subvalvular thickening as well as calcification. A score of ≤ 8 predicts a more favorable procedural, short, intermediate and long-term outcome (including survival). Other important predictors of procedural success and long-term outcome include commissural calcification or fusion, pre-procedure mitral regurgitation >2+, post-procedure mitral regurgitation >3+, age, prior surgical commissurotomy, NYHA functional class IV, and higher post-procedure pulmonary artery pressure (8, 9).

**Figure 3:**
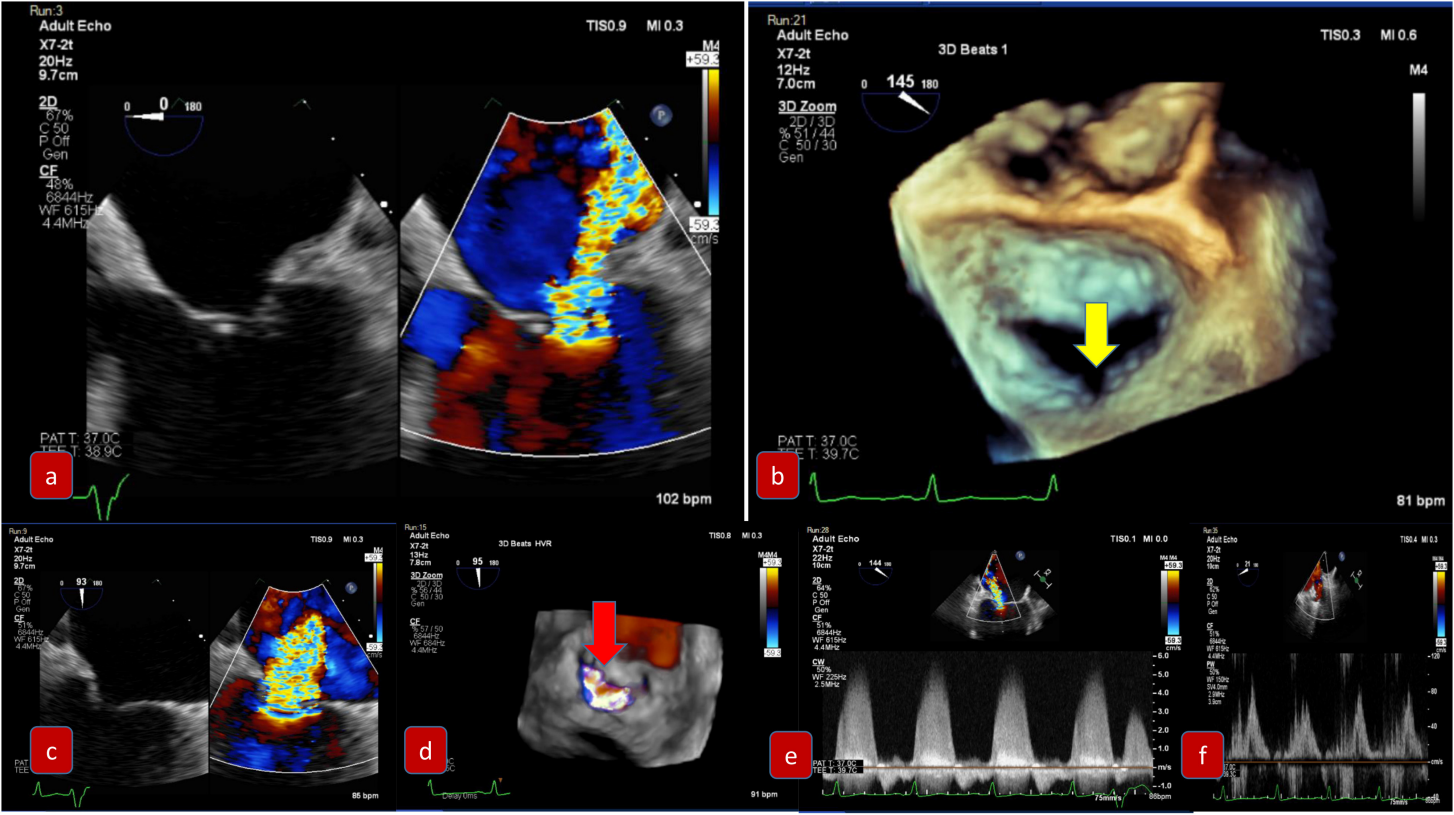
Pre-procedural evaluation for MitraClip: clockwise imaging: **(a)** mid-esophageal view 0 degrees demonstrating severe mitral regurgitation **(b)** 3D-TEE surgical view with a posterior cleft like indentation **(c)** mid-esophageal view 90 degrees—commissural view: severe mitral regurgitation **(d)** 3D-TEE with color demonstrating the regurgitation through the coaptation gap **(e)** continuous wave Doppler across mitral valve **(f)** pulmonary vein signal—pulsed wave Doppler.

**Figure 4:**
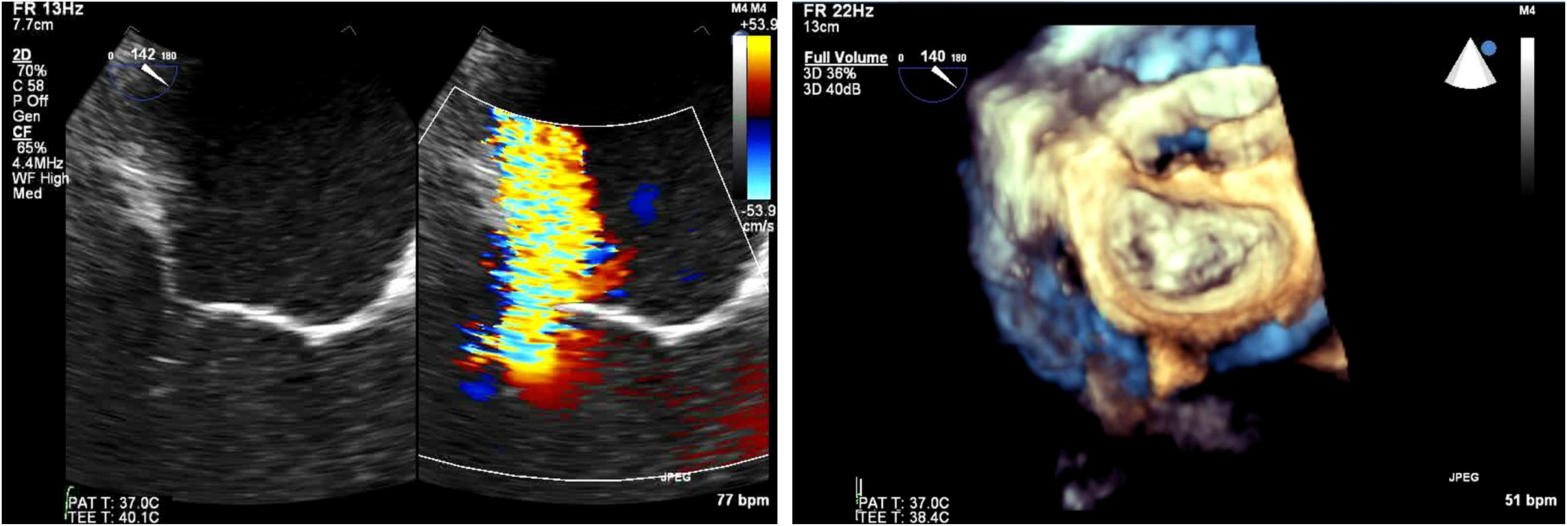
(Left) Tranesophageal echocardiography at 140 degrees for the evaluation of severe mitral regurgitation—(right) MitraClip positioning and 3D- transesophageal surgical view.

For mixed MVD or pure mitral regurgitation, the development of 3D TEE has allowed us the reconstruction of the mitral valve in a similar way as the surgeon would view this in the operating room (Figure 3). Another important feature and still a matter of debate is the identification of clefts and their differences to indentations, in myxomatous mitral disease. The deep cleft-like indentations are frequent on the posterior side of the mitral valve and are easily identified with 3D TEE—thus require closure during transcatheter valve repair (Figure 4). Anatomically, they are not related to excess annular enlargement or prolapse, butas an outcome of single scallop prolapse with paucity of the tissue and greater separation between the scallops (10).

(ii) Intra-procedural echocardiography

Care should be taken in the evaluation of MR during general anesthesia as the degree of regurgitation as well as Doppler values may be underestimated. The echocardiographic views differ depending on the intervention which is taking place.

### Intra-Procedural Echocardiography for Edge to Edge Repair

COAPT trial (11, 12) and MitraFR (13) trial had contradictory results but with a closer look, there were huge differences in the selection of patients for both studies, and specifically in effective orifice area (EROA) and left ventricular (LV) size. The differences between the two studies, assisted in the development of a new physiologic concept of proportional vs. disproportional mitral regurgitation (MR) (14, 15). This concept is based on the fact that effective regurgitant orifice area (EROA) depends on LV end-diastolic volume (LVEDV), ejection fraction (LVEF), regurgitant fraction, and the velocity-time integral of systolic mitral regurgitation. MitraClip is the first percutaneous device to be a part of the guidelines for management of severe symptomatic MR, with persistent heart failure symptoms despite rigorous guideline directed medical therapy (16).

For edge to edge repair, the bicaval view is important for the advancement of the steerable guide catheter while all TEE planes focusing on the mitral valve are important for the advancement of MitraClip in the left ventricle and the grasping of the leaflets. It is recommended to initially close the MitraClip only up to 60–90° and aim for a full closure of the Clip only after determination of proper leaflet insertion and demonstration of MR reduction. For assessment of proper leaflet insertion into the MitraClip multiple planes are useful. The insertion of the posterior leaflet is commonly best seen in the LVOT view, and the insertion of the anterior leaflet in the four-chamber view. The intercommissural view can add information such as entrapped chordae tendinae which may be visible at the free edges. Once the leaflets appear securely inserted and well-positioned in between the Clip arms and the grippers and some reduction in mitral regurgitation is documented, the MitraClip can be fully closed. An example of preprocedural imaging for MitraClip is demonstrated on Figure 3 while another example of positioning is demonstrated on Figure 4.

After MitraClip positioning, the evaluation of residual mitral regurgitation is important in deciding the number of clips to be employed. With implantation of a second MitraClip, the orientation of the second Clip should be optimized by 2D and 3D echocardiography in the left atrium. During advancement of the MitraClip from the left atrium into the left ventricle, the Clip should be closed to avoid any interference or entanglement with the chordae tendinae then re-opened in the ventricle. In general, fluoroscopy is more helpful than TEE for the positioning of a second MitraClip which should be aligned as parallel as possible to the first Clip. Folding of leaflet tissue between two MitraClips should be avoided as this may cause irreparable residual mitral regurgitation.

For color quantification (17, 18), the area of color jets may be larger with multiple jets, which commonly occur after a MitraClip is implanted (due to the addition of multiple jet areas), in comparison to a single jet. This may potentially lead to overestimating residual regurgitation in patients with multiple jets. In addition, artifacts caused by the MitraClip may also alter the interpretation. Nonetheless, small persistent color jets, even if multiple, are certainly congruent with mild mitral regurgitation. The study by Biaggi et al. (19) demonstrated that a post interventional transmitral gradient by continuous-wave Doppler of ≥5 mmHg best predicted elevated transmitral gradients at discharge.

### Transcatheter Mitral Valve Replacement and Paravalvular Leak Closure

**Figure 5:**
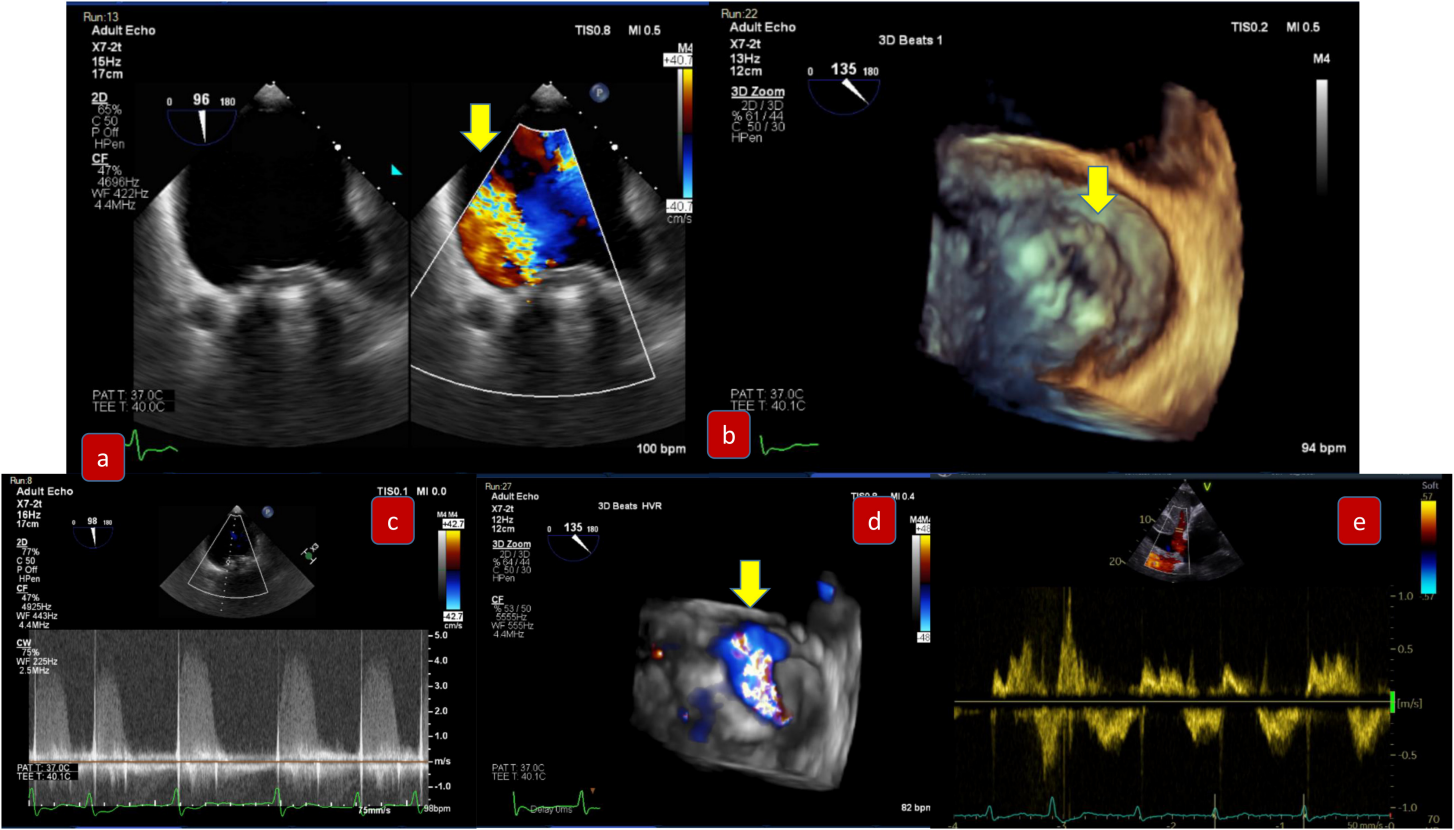
Pre-procedural evaluation for paravalvular leak due to degenerative rocking of a mitral prosthesis: **(a)** mid-esophageal view 90 degrees demonstrating severe paravalvular mitral regurgitation **(b)** 3D-TEE surgical view with slight detachment of the prosthetic valve from the suture line and posterolateral gap **(c)** Continuous wave Doppler of severe mitral regurgitation **(d)** 3D-TEE with color demonstrating the regurgitation **(e)** pulmonary vein signal—pulsed wave Doppler.

Transcatheter mitral valve replacement may be performed either trans- septal or trans-apical. TEE guidance to ensure the guide wire and device delivery system are centrally located (between A2 and P2) is important to avoid chordal rupture as well as to avoid interference with the anterior mitral leaflet and acute severe MR. After deployment of the device, multiplane 2D and 3D TEE are fundamental for device positioning, stability and function (transvalvular gradient and any residual mitral regurgitation). The imager should carefully identify central MR jets through the bioprosthesis due to cusp malfunction, which would be assessed with correct alignment of the continuous wave Doppler. Paravalvular leak (Figure 5) may result from suboptimal valve sizing and multimodality imaging plays a vital role in the correct sizing prior to the procedure. However, even with the right sizing, there may be inadequate seating due to insufficient capture of the native valve to which the prosthetic valve is anchored. Endocarditis or extremely calcified annulus may also be important causes of mitral PVL development.

Another important part of TMVR is to avoid LVOT obstruction. 3D TEE and CT software simulations add invaluable information in procedure planning, predicting the risk of post-operative LVOT obstruction. The LVOT size, aorta- LVOT angulation, the length of anterior mitral valve leaflet as well as the distance between the aorto-mitral continuity and basal ventricular septum should be monitored at all times during the mitral valve positioning and deployment procedure.

### (i) Post-procedural

**Figure 6:**
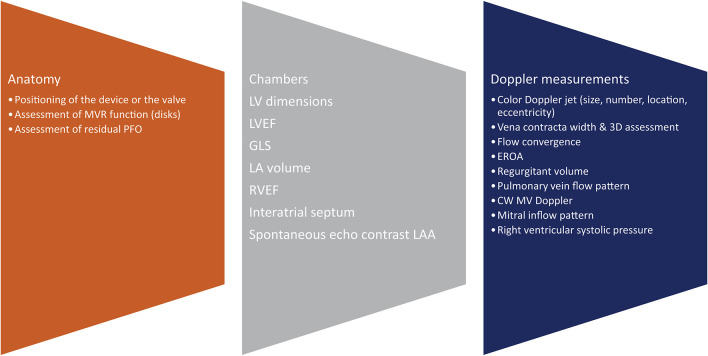
Post-procedural assessment. MVR, mitral valve replacement; PFO, patent foramen ovale; 3D, 3-dimensional; EROA, effective orifice area; CW, continuous wave; MV, mitral valve.

Each native valve has its own anatomical and physiological characteristics therefore, an individualized approach should be adopted (Figure 6). Color Doppler is of paramount importance in the assessment of residual MR, the number of jets and their orientation, and the integration with other parameters such as vena contracta, PISA, and regurgitant volume when appropriate. A change in the pulmonary vein reversal pattern may indicate mild mitral regurgitation post procedure. The same happens when rather than a prominent E wave, we depict a prominent A wave at the end of the procedure. Transmitral pressure gradient, LVOT obstruction and mitral valve area are also very important parameters that must be assessed.

**Figure 7:**
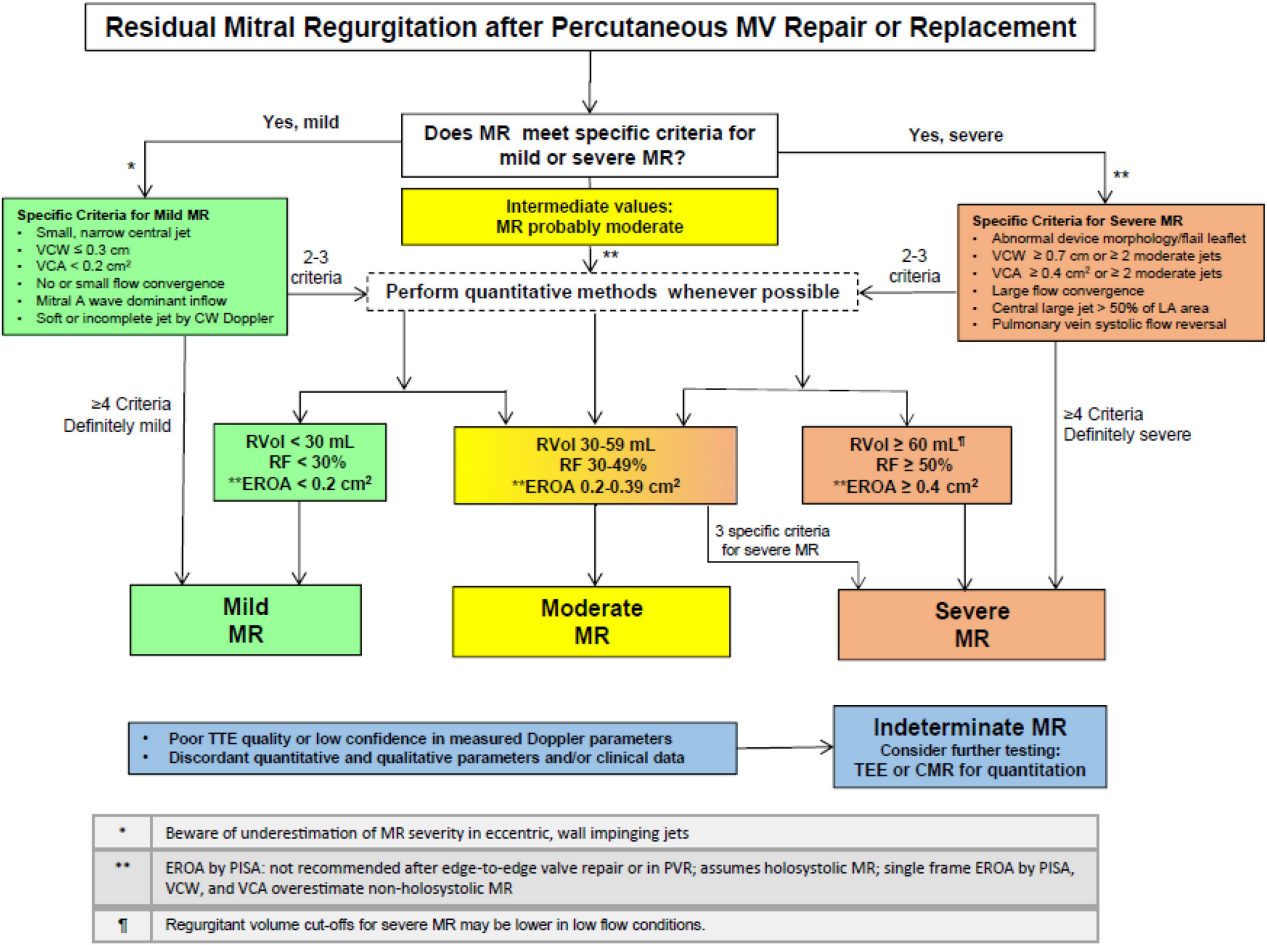
Algorithm to guide implementation of integration of multiple parameters of MR severity after mitral valve percutaneous interventions (with permission from JASE).

As a summary of the echocardiographic assessment post-procedurally, Dr. Zoghbi et al. (20) suggested an algorithm to guide the integration of multiple parameters of MR severity after mitral valve percutaneous interventions (Figure 7).

## Computed Tomography and Cardiac Magnetic Resonance

Computed tomography (CT) and cardiac magnetic resonance (CMR) are complementary tools to echocardiography that provide valuable information on mitral valvular structure, function, and associated chamber volumes (21, 22). CT and CMR have the advantage of improved spatial resolution compared to echocardiography, which is particularly helpful in mitral annular calcification (MAC), congenital heart disease, and post-surgical anatomy (23). With the rapid development of novel mitral valve interventions such as transcatheter mitral valve repair (TMV) and replacement (TMVR), multimodality imaging with CT and CMR plays a pivotal role in determining eligible candidates, pre-procedural planning and predicting complications (21, 24).

### Computed Tomography

Multidetector CT adds precision to planning the optimal intervention by determining annular size, landing zone characteristics, and predicting the risk of post-operative LVOT obstruction (24). Left ventricular outflow tract (LVOT) obstruction after transcatheter mitral valve replacement (TMVR) is associated with high mortality, but CT imaging can help predict the risk of this complication (25). Furthermore, pre-procedural cardiac CT can provide information on coronary anatomy, allowing a better procedural planning and estimation of patients' risk (26).

A common manifestation of mitral valve disease is severe MAC leading to mitral stenosis and mitral regurgitation (MR) (Figure 1). Surgery in patients with severe MAC has a high risk for complications, so TMVR is often preferable (27). CT can evaluate the extent of invasion of MAC and can guide prosthetic valve sizing by estimating changes to the LVOT. With TMVR, the mitral anterior leaflet migrates toward the interventricular septum creating a new LVOT (neo-LVOT), which is at risk of obstruction (25). Multiple CT measurements have been described in the literature. Praz et al. looked at 21 patients with severe MAC undergoing TMVR and reported that the CT measurement of Mitral Annular Area and Average Annular Diameter were reliable for valve selection, simulating the relative LVOT reduction, and predicting post-procedural gradients in the aorta (27). A recent study by Yoon et al. found that a neo-LVOT area cut-off of 1.7 cm^2^ had the greatest discriminatory value for predicting LVOT obstruction post TMVR (25). Further, software simulation is being developed to virtually implant the valve and anticipate dynamic interactions between the prosthesis and surrounding structures (28).

Patients not suitable for completely interventional TMVI in MAC, can be treated with the adoption of a hybrid technique, where a trans-catheter aortic valve is implanted in mitral position during open heart surgery. The advantages in this setting would be multiple. An extensive annular decalcification will not be needed, with the consequent reduction of the surgical risk; the trans-catheter valve will allow a greater EROA even in small mitral valve annuli with good hemodynamic results; the technique will be easily reproducible also in a minimally invasive setting. Most importantly, the possibility of surgically removing the anterior mitral leaflet during an open operation reduces drastically the risk of LVOT obstruction which is still matter of significant concern.

Complications due to mitral valve intervention are also well-suited for assessment by CT and CMR. CT has the advantage of increased spatial resolution and is less prone to artifact from metal and prosthesis artifact (23). Furthermore, it can identify paravalvular abscesses, restricted prosthesis excursion, valve dehiscence, and aneurysms (23). Coronary CT can also identify injury to the left circumflex artery which can occur from surgical injury to structures around the mitral annulus (23).

Coronary CT is valuable for preoperative planning in young patients. In this category of patients, the etiology of mitral valve disease is often myxomatous in nature and associated with mitral regurgitation secondary to valve prolapse. In this setting, coronary CT allows for pre-operative, non-invasive visualization of coronary arteries in low-risk patients (21).

### Cardiovascular Magnetic Resonance

**Figure 8:**
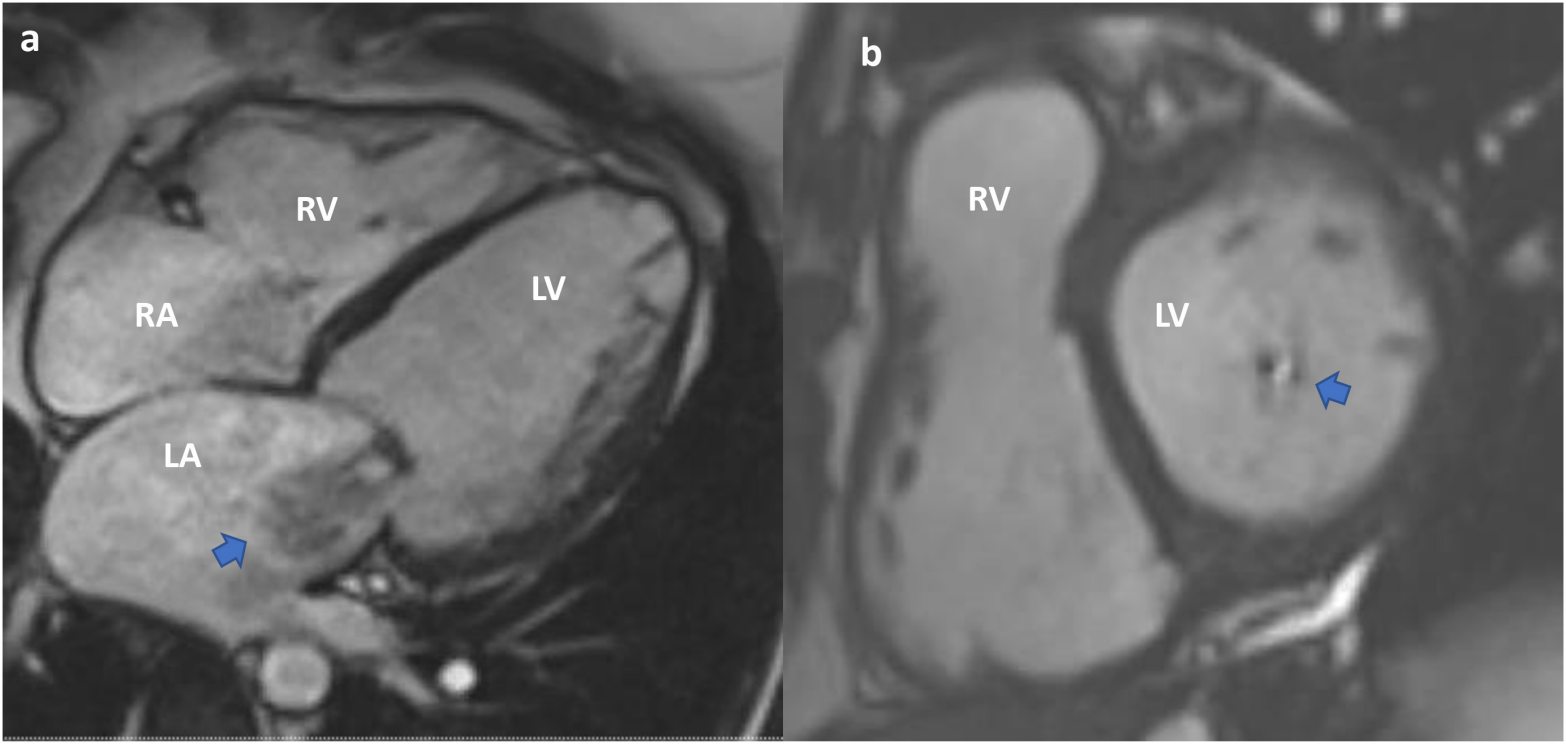
Cardiac MRI. Functional mitral regurgitation planning for mitral valve clip. **(a)** SSFP Cine 4-chamber showing severely dilated left ventricular cavity with “tented” mitral valve leaflets and evidence of significant mitral regurgitation (arrow) into a dilated left atrium. **(b)** SSFP Cine short axis of the mitral valve closure showing the jet of mitral regurgitation mostly confined toward the posterior annulus (arrow).

**Figure 9:**
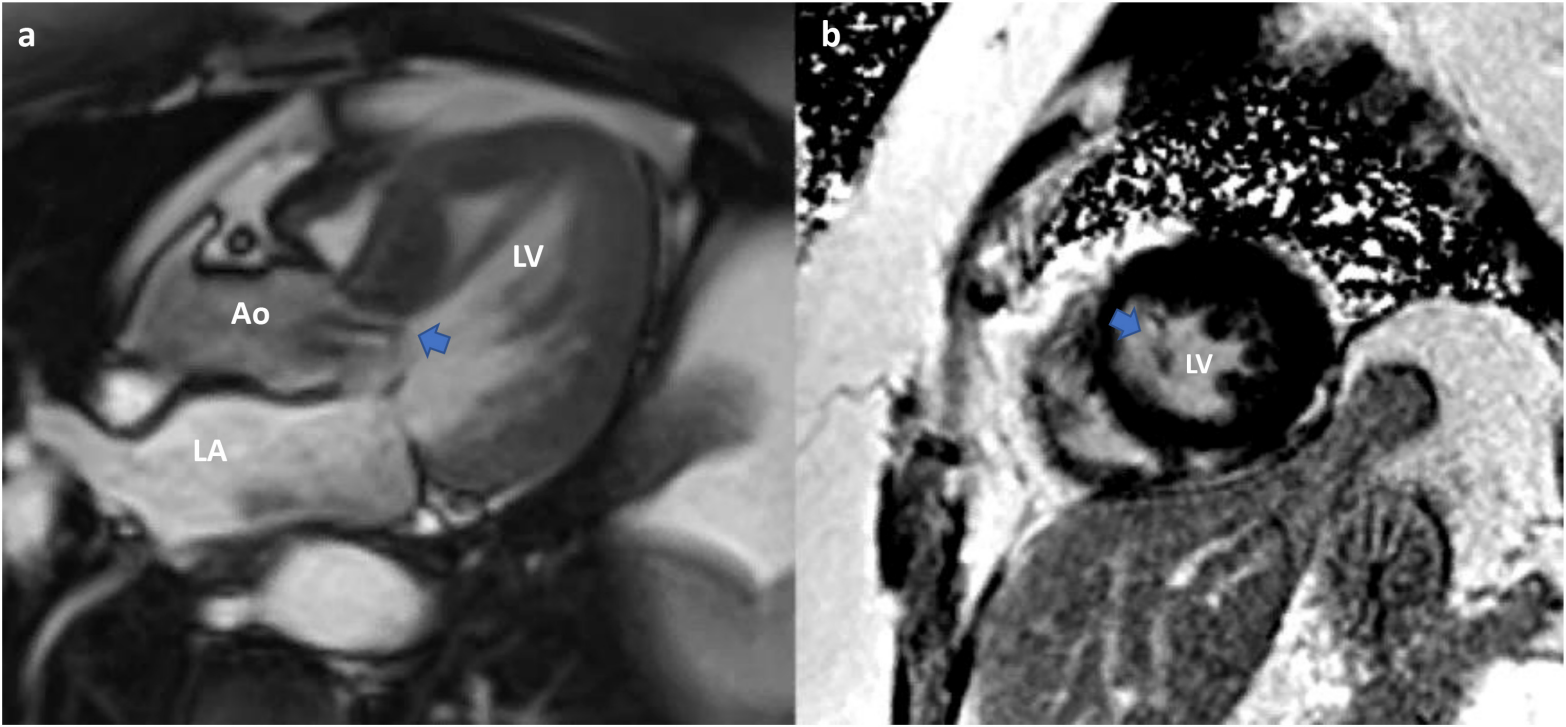
Cardiac MRI. Planning of alcohol septal ablation in patient with hypertrophic cardiomyopathy and severe mitral regurgitation due to systolic movement of the anterior mitral valve leaflet (SAM). **(a)** SSFP-cine Three chamber: Systolic motion of the anterior MV with intracavitary flow acceleration (diphasic) and consequent significant mitral regurgitation. Asymmetrical septal hypertrophy. **(b)** T1-weighted late gadolinium enhancement (LGE-MRI) Short axis mid ventricular showing late gadolinium hyperenhancement of the papillary muscle-chordal apparatus (arrow) due to fibrosis secondary to traumatic contact with the septum.

CMR provides valuable functional data and allows accurate quantification of chamber volumes. It is particularly helpful in quantifying MR (29) (Figures 8, 9). CMR is indicated for MR when echocardiography images are suboptimal, when there is discordance between 2D and Doppler echo findings, or when there is a discordance between clinical and echocardiographic findings (30). CMR has a better ability to quantify regurgitant volumes compared to 2-dimensional echocardiography, which often underestimates the regurgitant volume (28). CMR quantification is not influenced by the jet shape or direction, and has better inter-observer consistency (21). As recommended by the current guidelines, the most widely used CMR method to quantify MR is to measure the difference between total LV ejection volume and aortic forward flow (7, 21, 31).

In cases of severe MR, CMR can provide guidance on the optimal time to intervene (7). CMR is more sensitive to changes in LV chamber size compared to echocardiography (7). Moreover, in cases of ischemic MR, CMR can elucidate the degree of ischemia and provide guidance on the reversibility of MR post revascularization (21).

While CMR has good concordance with echocardiography to localize leaflet pathology in mitral valve prolapse, an important limitation is the suboptimal assessment of small structures such as chordae or vegetations due to the partial volume effect from limited CMR resolution of finer voxels (21).

As a conclusion, while echocardiography remains the cornerstone imaging methodology, integrated CT and CMR are increasingly becoming the standard of care in mitral valve assessment prior to intervention. This provides operators with the best chance of procedural success.

### Hybrid Imaging and 3D Printing

The future of imaging lays on hybrid imaging which fuses the advantages and overcomes the disadvantages of all the above mentioned imaging modalities. Pascual et al. (32) described the value of hybrid imaging in transcatheter interventions. Furthermore, 3D printing may also have a great value in challenging anatomy such as congenital heart disease or complex valvular pathology (33).

### Gaps in Knowledge and Future Perspectives

As CT and CMR gain more wide-spread use, it will be important to standardize protocols and evaluate the most reliable measurements. To this end, Garg et al. recently published a consensus statement supporting a standardized CMR protocol which includes the assessment of MR (30). Future directions in CMR assessment include the development of 4D-flow techniques that improve functional assessments of smaller valvular lesions. 4D-flow shows promise in enhancing precision of MR quantification, but further studies looking at clinical outcomes are needed (30, 34).

While CT assessment provides a high degree of precision, it comes at the cost of radiation and risk of contrast injury. Decreasing the radiation associated with CT and the time required for obtaining the optimal image are future directions of research which will allow us to more safely provide CT assessment of mitral valve pathology. Moreover, contrast-associated renal injury remains an important concern given its association with increased mortality, so further studies in reducing contrast injury are needed (31).

Finally, an exciting area of research is in the hybrid modality approach (34). For example, van Rosendael et al. looked at 73 patients referred for transcatheter aortic valve implantation (TAVI) who also had MR and were assessed with combined CT and echocardiography to evaluate MR (35). An integrated regurgitant volume parameter was measured by combining the true cross-sectional mitral regurgitant orifice area from CT with the velocity time integral of the MR jet taken from echocardiography (35). With this method, 10% of the patients were reclassified from severe to non-severe MR and 14% from non-severe to severe (35). This approach may allow clinicians to utilize the best of each modality.

### Future Directions of Transcatheter Interventions

Transcatheter mitral valve interventions have overcome multiple limitations and have a bright future: the future is standing on the rapid development of imaging and the greater application of hybrid modalities. Furthermore, transcatheter procedures will expand in areas beyond the current guidelines such as in dilated left ventricles, (36). We also witness the continuous expansion of indications for mitral valve interventions in mitral clefts or congenital heart disease (37). In addition, technology will help in making new customized prosthetic valves available for complex anatomy patients (2).

## Conclusion

As a conclusion, TMVI multimodality imaging is expanding fast and in all dimensions. New valves and devices, but also large studies with long term results of the existing one, will shed light in the management of mitral valve pathology.

## Author Contributions

All authors listed have made a substantial, direct and intellectual contribution to the work, and approved it for publication.

## Conflict of Interest

The authors declare that the research was conducted in the absence of any commercial or financial relationships that could be construed as a potential conflict of interest.

## Abbreviations

EROA: Effective regurgitant orifice area
LVEF: Ejection fraction
LV: Left ventricular
LVEDV: Left ventricular end-diastolic volume
LVOT: LV outflow tract
MR: Mitral regurgitation
TTE: Transthoracic
TEE: Transesophageal echocardiography
TMVI: Transcatheter mitral interventions.
